# Mild therapeutic hypothermia upregulates the O-GlcNAcylation level of COX10 to alleviate mitochondrial damage induced by myocardial ischemia–reperfusion injury

**DOI:** 10.1186/s12967-024-05264-x

**Published:** 2024-05-22

**Authors:** Wei Deng, Yixuan Chen, Jing Zhang, Jitao Ling, Zhou Xu, Zicheng Zhu, Xiaoyi Tang, Xiao Liu, Deju Zhang, Hong Zhu, Haili Lang, Lieliang Zhang, Fuzhou Hua, Shuchun Yu, Kejian Qian, Peng Yu

**Affiliations:** 1 Department of Anesthesiology, The Second Affiliated Hospital, Jiangxi Medical College, Nanchang University, 1st Minde Road, Nanchang, Jiangxi province 330006 China; 2 Department of Endocrinology an Metabolism, The Second Affiliated Hospital, Jiangxi Medical College, Nanchang University, 1st Minde Road, Nanchang, Jiangxi province 330006 China; 3 Food and Nutritional Sciences, School of Biological Sciences, The University of Hong Kong, Pokfulam Road, Hong Kong, China; 4 Department of Cardiology, Sun Yat-Sen Memorial Hospital of Sun Yat-Sen University, Yanjiang Road, Guangzhou, Guangdong Province China; 5 Jiangxi Key Laboratory of Neurological Tumors and Cerebrovascular Diseases, Nanchang, Jiangxi province China; 6 Jiangxi Health Commission Key Laboratory of Neurological Medicine, Nanchang, Jiangxi province China; 7 Department of Intensive Care Unit, The First Affiliated Hospital, Jiangxi Medical College, Nanchang University, Nanchang, Jiangxi province China; 8 The Second Clinical Medical College, Jiangxi Medical College, Nanchang University, Nanchang, Jiangxi province 330006 China

**Keywords:** MTH, O-GlcNAcylation, OGT, COX10, Mitochondrion

## Abstract

**Objective:**

Mild therapeutic hypothermia (MTH) is an important method for perioperative prevention and treatment of myocardial ischemia–reperfusion injury (MIRI). Modifying mitochondrial proteins after protein translation to regulate mitochondrial function is one of the mechanisms for improving myocardial ischemia–reperfusion injury. This study investigated the relationship between shallow hypothermia treatment improving myocardial ischemia–reperfusion injury and the O-GlcNAcylation level of COX10.

**Methods:**

We used in vivo Langendorff model and in vitro hypoxia/reoxygenation (H/R) cell model to investigate the effects of MTH on myocardial ischemia–reperfusion injury. Histological changes, myocardial enzymes, oxidative stress, and mitochondrial structure/function were assessed. Mechanistic studies involved various molecular biology methods such as ELISA, immunoprecipitation (IP), WB, and immunofluorescence.

**Results:**

Our research results indicate that MTH upregulates the O-GlcNACylation level of COX10, improves mitochondrial function, and inhibits the expression of ROS to improve myocardial ischemia–reperfusion injury. In vivo, MTH effectively alleviates ischemia–reperfusion induced cardiac dysfunction, myocardial injury, mitochondrial damage, and redox imbalance. In vitro, the OGT inhibitor ALX inhibits the OGT mediated O-GlcNA acylation signaling pathway, downregulates the O-Glc acylation level of COX10, promotes ROS release, and counteracts the protective effect of MTH. On the contrary, the OGA inhibitor ThG showed opposite effects to ALX, further confirming that MTH activated the OGT mediated O-GlcNAcylation signaling pathway to exert cardioprotective effects.

**Conclusions:**

In summary, MTH activates OGT mediated O-glycosylation modified COX10 to regulate mitochondrial function and improve myocardial ischemia–reperfusion injury, which provides important theoretical basis for the clinical application of MTH.

## Introduction

Ischemic cardiomyopathy is one of the leading causes of death worldwide and the second leading cause of mortality in China [6, 7, 15] (Cw et al., 2023, 2022; G et al., 2023). Percutaneous coronary intervention, coronary artery bypass grafting, and other reperfusion methods are currently the most successful methods for treating ischemic cardiomyopathy clinically [30, 38] (Ly et al., 2020; R et al., 2010). However, these interventions may exacerbate myocardial injury, eliciting myocardial ischemia–reperfusion injury (MIRI) [8] (Dj and Dm, 2013). MIRI stands as a leading cause of mortality in patients afflicted with ischemic cardiomyopathy and coronary heart disease. It also represents a frequent complication arising during anesthesia and surgical procedures. Ischemic preconditioning (IPC) and pharmacological preconditioning (PPC) are the main means to improve myocardial ischemia–reperfusion injury in clinical practice [20]. However, due to the limited use conditions, the two pretreatment methods mentioned above cannot have a positive clinical effect. In a clinical study, the researchers pointed out that remote ischemic preconditioning did not improve the clinical outcomes of patients who received elective on-pump coronary artery bypass surgery (with or without valve surgery) [9] (Dj et al., 2015). New researches indicate that the new nano silver particles have good antibacterial and anti-cellular oxidation effects, but whether they have therapeutic effects on perioperative myocardial ischemia–reperfusion injury is still unknown [2, 52]). Therefore, more effective treatments are imperative.

In 1997, Sessler first proposed the concept of mild perioperative hypothermia in the New England Journal of Medicine, demonstrating its ability to prevent cerebral ischemia when the core temperature is maintained at 33–35 °C [46]. Follow-up clinical studies conducted in 2002 provided additional evidence that therapeutic mild hypothermia significantly lowers both mortality rates and the extent of neurological damage after cardiac arrest [45] (Sa et al., 2002). Moreover, studies have shown that hypothermia administered during emergency percutaneous coronary intervention mitigates infarct size and attenuates MIRI (S et al., 2008). By preliminary research, we have demonstrated that mild hypothermia therapy can significantly lessen rat myocardial ischemia–reperfusion injury. Additionally, numerous studies have confirmed that mild hypothermia therapy can improve mitochondrial function by inhibiting mitochondrial autophagy, thereby improving myocardial ischemia–reperfusion injury [19, 22, 31, 36, 42] (J et al., 2014; M et al., 2019; R et al., 2013; S et al., 2019). However, the mechanisms underlying hypothermia-mediated mitochondrial autophagy regulation remain poorly understood.

Post-translational modification (PTM) of proteins is a fundamental regulatory mechanism that influences protein activity, localization, expression, and interaction with other cellular components by appending or removing specific moieties on protein amino acid residues [40, 43] (Rg and F, 1993; W. S et al., 2022). A growing body of research has unveiled that numerous critical biological processes and diseases are influenced not solely by protein abundance but also by a variety of PTMs [24] (Z and J, 2022). O-linked GlcNAc acylation constitutes a form of post-translational protein modification [51] (X and K, 2017). The O-GlcNAc modification cycle is actively regulated by a duo of enzymes, consisting of O-GlcNAc transferase (OGT) and O-GlcNAc enzyme (OGA). O-GlcNAcylation plays an important role in organic diseases and cancers. Research has found that the level of O-GlcNAcylation is closely related to the occurrence of heart failure, and an excess of O-GlcNAcylation will cause cardiovascular disease [37] (P et al., 2021). Concurrently, O-GlcNAcylation activates PKG1, facilitating lactate release, and also prompts the translocation of PKG1 into mitochondria. This dual effect ultimately promotes the proliferation and growth of colon cancer cells [18] (H et al., 2020). Notably, studies have shown that glycosylation plays a crucial role in myocardial ischemia–reperfusion injury [16, 17, 25, 49] (Ga et al., 2009; K et al., 2011; V et al., 2007), especially in governing mitochondrial homeostasis [44, 48] (V et al., 2008). O-GlcNAcylation affects ETC complexes and TCA circulating enzymes by modifying mitochondrial proteins, improving mitochondrial function, and reducing ROS release, thereby exerting myocardial protective effects [21] (D. J et al., 2022).

Mitochondrial damage and mitochondrial dysfunction are the most common causes of perioperative organ damage [1, 10], and it is worth noting that COX10 plays a crucial role in them (D. D. L et al., 2023). COX10 is an assembly factor of Cytochrome c oxidase [57] (Z et al., 2010). Research findings have indicated that hypoxia has the potential to downregulate COX10 expression, thereby exacerbating mitochondrial damage and injury (Z et al., 2010). Furthermore, investigators have observed that mild hypothermia significantly diminishes the release of Cytochrome c 5 h after an ischemic event [33] (Ma et al., 2002). However, these two studies did not clarify how MTH regulates COX10 to alleviate myocardial injury. Importantly, studies have shown that the glycosylation level of the mitochondrial DNA encoded subunit I of complex IV (COX I) significantly affects mitochondrial function. When significantly elevated, it worsens myocardial damage and mitochondrial deterioration [55] (Y et al., 2009). Therefore, we propose a hypothesis that mild hypothermia therapy may improve mitochondrial function by modulating the O-GlcNAcylation status of COX10 O-GlcNAcylation, ultimately alleviating myocardial ischemia–reperfusion injury. We validated the effects of MTH on mitochondrial function and ROS levels through immunofluorescence, electron microscopy, and other techniques. At the same time, we explored the impact of COX10 on O-GlcNAcylation levels through research methods such as immunofluorescence, IP, and WB to clarify the important role of mitochondrial damage in myocardial ischemia–reperfusion.

## Methods

### Animal treatment and cell culture

Healthy adult male Sprague–Dawley rats were housed under a controlled environment with a 12-h light/12-h dark cycle and maintained at a temperature of 25 ± 1 °C. The animal experiments conducted in this study received approval from the Animal Care and Use Committee of the Second Affiliated Hospital of Nanchang University. Rats were randomly divided into three groups: Sham group, no treatment; the IR + 37 °C group, which was subjected to ischemia for 30 min and then reperfusion for 120 min at 37 °C; IR + 34 °C group, the MTH (34 °C) was induced during the reperfusion period.

To acquire cells for research purposes, the heart was initially sectioned into small 1mm^3^ fragments. These fragments were then meticulously placed within a centrifuge tube, along with 5 ml of digestive solution. The mixture was then digested at 37 °C for 5 min, after which the supernatant was removed. An additional 5 ml of digestive solution was then added, and the mixture was subjected to a 20-min digestion process, followed by one minute of blowing with a straw. The remaining undigested heart fragments were subsequently aspirated into another centrifuge tube. To halt the digestion process, 2 ml of cold culture medium was added, and the mixture was centrifuged at 1000 rpm for 5 min. Once more, the supernatant was extracted, and the sediment was resuspended in 2 ml of D-Hanks solution. The mixture was once again centrifuged at 1500 rpm for 10 min, and the supernatant was once more removed. Finally, 2 ml of culture medium was added to the sediment, and the mixture was blown with a straw to create a cell suspension. This process was repeated by adding 5 ml of digestive solution to the undigested heart fragments, followed by digestion and centrifugation. The cell suspension was then merged and placed in a carbon dioxide incubator (37 °C, 5% CO2) for cultivation.

The cells were divided randomly into five groups for the experiment. The first group was the control group which received no treatment. The second group, HR + 37 °C + Veh, was exposed to hypoxia for 120 min followed by 120 min of reoxygenation at 37 °C. The third group, HR + 37 °C + ThG, was subjected to hypoxia for 120 min, followed by 120 min of reoxygenation at 37 °C, with the addition of O-GlcNAcase (OGA) inhibitor ThG 0.5 μM during reoxygenation. The fourth group, HR + 34 °C + Veh, underwent MTH (34 °C) induction during the reperfusion period. Finally, the fifth group, HR + 34 °C + ALX, underwent a 120-min period of hypoxia, followed by 120 min of reoxygenation at 34 °C with the addition of the OGT inhibitor ALX at a concentration of 10 μM during reoxygenation.

### Construction of Langendorff isolated heart perfusion model, cell model establishment, and hemodynamic detection

**Fig.1:**
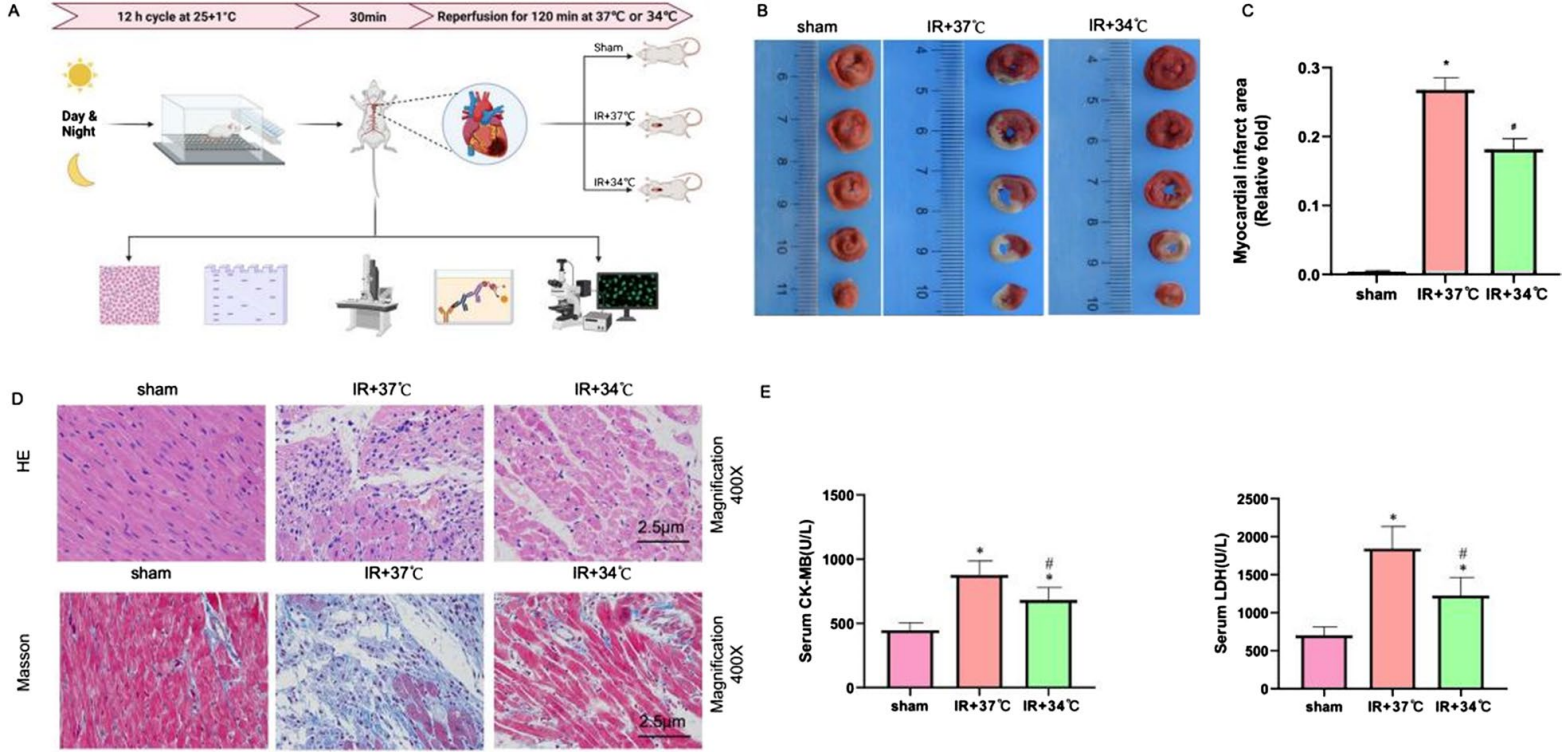
MTH reduces myocardial ischemia–reperfusion injury. **A** Flow Chart for Building Animal Models. **B** TTC staining. **C** Statistical map of the proportion of myocardial infarction area. **D** Statistical map of the proportion of myocardial infarction area. **E** Serum myocardial injury marker concentration (CK-MB & LDH). *P < 0.05 VS sham group; #P < 0.05 VS IR + 37 °C group

Healthy adult male Sprague–Dawley rats were anesthetized using 3% pentobarbital sodium (50 mg/kg) and were then injected intraperitoneally with heparin(1000 IU/kg, each n = 8). After midline thoracotomy, the heart was excised within 1 min and the aorta was cannulated for retrograde perfusion at a constant pressure of 80 mmHg by a Langendorff device with a modified Krebs–Henseleit (MKH) buffer. The buffer was continuously gassed with a mixture of 95% oxygen and 5% carbon dioxide to ensure optimal oxygenation. The temperature of the buffer was regulated at either 37 ± 0.2 °C or 34 ± 0.2 °C using a circulating water system. The initial left ventricle end-diastolic pressure (LVEDP) was set to 10 mmHg by a latex balloon filled with bubble-free saline and Med Lab 6.0 software (ZSDiChuang Science and Technology Development Co Ltd, Beijing City, China) was used to record hemodynamic changes (except for the sham group), including heart rate (HR), left ventricular systolic pressure (LVSP), LVEDP and ± dp/dtmax. Rats with frequent arrhythmias, refractory ventricular fibrillation, HR < 180 beats/min, or LVSP < 75 mmHg were excluded. The construction method of Langendorff’s in vitro myocardial model refers to previous articles published by our research team (Zhang et al., 2020). The experimental process is shown in Fig. 1 A.

The myocardial cell H/R model was established using the ischemia–reperfusion liquid culture method. According to the instructions, hypoxia liquid (blood deficiency) and reoxygenation liquid (reperfusion liquid) were prepared. Replacing the normal culture medium with anoxic solution is anoxia, and then replacing the anoxic solution with reoxygenation solution is reoxygenation. Based on preliminary experimental results, a hypoxia period of 2 h followed by a reoxygenation period of 2 h was determined as the optimal protocol to create the hypoxia/reoxygenation mode.

### Determination of myocardial infarct size.

After 2 h of reperfusion, the hearts were transferred to phosphate buffer solution (PBS, pH 7.4, maintained at 4 °C) and then frozen at −20 °C for 80 min. Each heart was cut into five cross sections using a blade, and the sections were incubated in 1% TTC prepared by dissolving1g of 2,3,5-triphenyltetrazolium chloride triazole (Sigma, T8877) into 100 mL of phosphate-buffered saline at 37 °C for 20 min. The hearts were fixed in 4% polyformaldehyde overnight after incubation. The myocardial infarct area changed to white, while the area at risk remained red. An Epson scanner (Seiko Epson Corporation, Suwa, Nagano Japan) was used to scan each slice, and thepercentage of myocardial infarction area divided by the total risk area was calculated by Image Pro Plus software.

### Hematoxylin and eosin (H&E) staining and Masson staining

H&E staining: the myocardial tissue obtained from rats was fixed in 10% paraformaldehyde, followed by embedding in paraffin and cutting into 4 µm sections. These sections were dewaxed with xylene twice (5 min each), and hydrated with 95% ethanol. After that, hematoxylic acid was added for 10 min and the section was then rinsed with tap water, differentiated with 1% HCl ethanol for 10 s and finally stained with 5% eosin for 1–3 min. The stained sections were dehydrated with ethanol for 25 min, dewaxed with xylene, sealed and dried with neutral sesin, and finally observed with a Leica IX71 microscope (× 400).

Masson staining: rat myocardial tissue was first rinsed with 4 °C physiological saline to remove any debris or impurities. Next, the tissue was fixed in a 4% tissue cell fixative and embedded in paraffin. The paraffin-embedded tissue was then cut into thin 4 µm sections. Dewaxing was performed using xylene I and xylene II for 10 min each. Subsequently, hydration was achieved through ethanol solutions of varying concentrations. The sections were then soaked in double-distilled water for 5 min. They were placed in Bouin solution for staining, rinsed with distilled water, and stained according to the instructions provided by the enhanced Masson trichrome staining kit. After rinsing the stained slices with ethanol solutions of different concentrations, they were made transparent using xylene three times. Finally, observation was conducted using a Leica IX71 microscope at a magnification of × 400.

### Determination of cardiac enzymes and oxidative stress damage markers

Whole blood of the rats was collected and centrifuged to separate serum. The contents of serum central muscle enzymes (CK-MB and LDH) and oxidative stress damage markers (MDA, SOD and GPX) were measured by ELISA. All steps were performed in strict accordance with the instructions.

### Detection of intracellular ROS

ROS production was evaluated by DHE staining in vivo and and DCFH-DA staining in vitro. DHE-staining: Slice myocardial tissue (10 μm) after being placed on a cationic anti-detachment glass slide, using a concentration of 5 μmol/L superoxide anion fluorescent probe solution of dihydroethylpyridine (DHE) was dropped onto tissue slices. Then heart slices were incubated at 37 °C in the dark for 30 min. Finally, wash the tablets twice with 0.01 mol/L PBS for 5 min per time, and seal them with the water-soluble solution. Finally, the heart slices were observed using a fluorescence microscope (Olympus IX53, Tokyo, Japan). Remove the cell culture medium and add DCFH-DA (5 um/mol) diluted to an appropriate volume. Incubate in a 37 °C cell culture incubator for 30 min. Wash cells three times with serum-free cell culture medium to fully remove DCFH-DA that has not entered the cells. And fluorescence intensity was measured using flow cytometry (Attune NxT). The experimental methods for these ROS assessments were based on the research conducted by Hu et al.[19].

### Mitochondrial function detection and electron microscopy observation

Myocardial tissue was taken and washed with 0.1 M sodium bicarbonate to remove any debris or impurities. Subsequently, the tissue was fixed in 1% OsO4 (Osmium tetroxide) solution for 1 h, then dehydrate in graded acetone and embed in Epon Araldite resin. Sections were stained with 2% Uranyl acetate and Renault Lead citrate, and observed using an electron microscope [58] (Zhu et al., 2021).

In addition to electron microscopy, Seahorse XF Cell Mito Stress Test Kit (catalog number 103015–100, Agilent, Delaware, USA) was used to detect mitochondrial respiratory capacity. The Seahorse XF Cell Mito Stress Test Kit was specifically designed to measure the oxygen consumption rate (OCR) using an Agilent Seahorse Xfe96 Extracellular Flux Analyzer (Agilent, Delaware, USA). All experimental steps shall be strictly carried out in accordance with the instructions.

### Immunoprecipitation

To lyse the cells, NP-40 lysis buffer was used on ice for 30 min. After cell lysis, the lysate was centrifuged to separate the supernatant. For immunoprecipitation (IP), 2 μg of specific antibodies was added to the supernatant, and protein-A/G + agarose gel was used to capture the antibody-protein complexes. Incubating it overnight in a shaker at 4 °C, then washing it sequentially with low salt NP-40 lysis buffer and high salt lysis buffer. After boiling for 10 min, the released protein was graded in 12% SDS-PAGE gel.

### Detection of cell activity and cytotoxicity

Cell viability was measured by MTT Cell Viability Assay kit and cytotoxicity was measured by LDH Cytotoxicity Assay Kit. All steps were performed in strict accordance with the instructions [53] (Xu et al., 2023b).

### Cell apoptosis detection

Cells were digested with 0.25% trypsin without EDTA. After digestion was terminated, the treated cells were collected and centrifuged at 1500 rpm for 5 min. The supernatant was removed, and was resuspended with PBS. Before using the membrane associated protein V-FITC/PI cell apoptosis detection kit for detection, we washed the cells twice with PBS at 1500RPM for 5 min. All steps were performed in strict accordance with the instructions. Finally, the level of apoptosis was observed by flow cytometry.

### Western blot

The total proteins from cell samples or hearts were extracted and homogenized by using the Total Protein Extraction Kit (P1250, Applygen Technologies, Beijing, China) as per the manufacturer's instruction. After centrifuging at 2000 × g for 5 min, the clarified supernatant was separated from the sample. Then we used BCA reagent kit (23227, Thermo, MA, USA) to quantify protein concentration according to the instructions. Protein was separated on 8% sodium dodecyl sulphate–polyacrylamide gel electrophoresis (SDS-PAGE) and then transferred onto a polyvinylidine fluoride (PVDF) membrane. Next, the PVDF membrane was blocked in 10% no-fat milk for 2 h and then incubated with primary antibodies of DNP, O-GlcNAc, OGT, OGA, GFAT and COX10, respectively, at 4°C overnight, followed by incubating with horseradish peroxidase-conjugated goat secondary antibody (1:1000, in 5% no-fat milk) at room temperature for 2 h. The same membrane was probed with anti-GAPDH or anti-Porin to control lane loading.

### Immunofluorescence (IF) staining

The cells were fixed in 4% Paraformaldehyde, permeated with 0.1% Triton X-100, and then incubated at 4 °C overnight with primary antibodies against O-GlcNAc and COX10. Then, the cells were incubated with CL594 coupled anti-rabbit (1:500, SA00013-4, Proteintech) or CL488 coupled anti-mouse IgG (1:500, SA00013-1, Proteintech) secondary antibodies, and the nuclei were stained with DAPI. Finally, the immunofluorescence-stained cells were imaged using an Olympus BX51 immunofluorescence microscope.

### Statistical methods

The data were shown as mean ± SD. One-way analysis of variance (ANOVA) was used to compare the data among the groups and used Welch correction to correct the P-value. P values < 0.05 were considered statistically significant. Statistical analysis was conducted using GraphPad Prism software (version 9.5) [11].

### Literature selection

In the process of writing the paper, we mainly rely on the PubMed database and use keyword searches to find the information we need. The main theme words include MTH, O-GlcNAcylation, OGT, COX10, Mitochondrion, MIRI, etc. At the same time, the period we searched is 1950–2024.

## Result

### MTH reduced myocardial infarction area and improved hemodynamic performance after ischemia–reperfusion injury in rats

Figure 1 reveals our assessment of myocardial infarction volume using TTC staining. Our findings indicated that in the IR + 37 °C group, the myocardial infarction volume ratio was approximately 26%, whereas in the IR + 34 °C group, the infarction volume ratio decreased significantly to 18% (see Fig. 1B, C). Additionally, we conducted histological examinations using HE and Masson staining, as depicted in Fig. 1D. The results showed that the arrangement of myocardial cells in the Sham group was regular, with obvious nuclei and no inflammatory cell infiltration. In IR + 37 °C group, the heart of mice displayed disordered central muscle cells, extensive myocardial cell necrosis, significant expansion of cell gap, and obvious myocardial fibrosis, with only a few surviving myocardial cells. In the IR + 34 °C group, we observed a relatively orderly arrangement of myocardial cells, along with a notable reduction in the extent of cell necrosis and fibrosis, both in terms of degree and scope. Furthermore, the myocardial zymogram results showed that MTH significantly reduced the levels of CK-MB and LDH in serum compared with the IR + 37 °C group (Fig. 1E, P < 0.05).

In addition, we also recorded the impact of MTH on hemodynamic parameters during myocardial ischemia–reperfusion injury in rats. The study found no significant differences in HR, LVSP, LVEDP, and ± dp/dtmax between the groups at baseline. However, significant differences were observed in HR, LVSP, LVEDP, and ± dp/dtmax (P < 0.05) among the groups during reperfusion at 30 min (T1), 60 min (T2), 90 min (T3), and 120 min (T4). Moreover, compared to the IR + 37 group, HR, LVSP, and ± dp/dtmax in the IR + 34 group significantly increased at all periods during reperfusion, while LVEDP significantly decreased (P < 0.05). Through observation, we found that the heart rate of the mice in the Sham group was about 260 beats per minute, while at T4, IR + 37 °C was only half of normal (P < 0.05). At T4, the LVEDP of the IR + 37 °C group was 7.5 times higher than that of the sham group (P < 0.05), and the LVEDP of the IR + 34 °C group was 5 times higher than that of the sham group (P < 0.05). The results are shown in Table 1. The experimental results showed that MTH protected against myocardial ischemia–reperfusion injury in rats, which was consistent with our previous research (Z. J et al., 2022; [35]).

**Table 1:** Myocardial haemodynamics during experiments

	Reperfusion				
Group	Baseline (T0)	30 min (T1)	60 min (T2)	90 min (T3)	120 min (T4)
HR (min-1)					
Sham group	267.19±17.07	262.28±8.18	261.52±21.68	268.58±15.25	263.18±22.15
IR+37 °C group	264.11±10.51	209.82±11.88*	189.26±16.11*	168.42±8.73*	132.90±12.56*
IR+34 °C group	266.43±15.24	242.00±9.14*#	219.45±22.52*#	207.99±10.33*#	195.98±14.69*#
LVEDP (mmHg)					
Sham group	7.13±0.19	7.34±0.32	7.40±0.79	6.90±0.59	5.67±0.46
IR+37 °C group	7.38±0.51	15.26±1.11*	27.21±1.13*	36.00±1.08*	42.73±0.74*
IR+34 °C group	7.09±0.35	10.03±1.04*#	22.50±1.08*#	25.87±3.24*#	30.51±1.46*#
LVSP (mmHg)					
Sham group	92.36±8.12	97.94±10.38	95.81±12.29	101.41±6.14	95.49±8.85
IR+37 °C group	96.61±9.47	79.42±3.57*	59.15±3.83*	45.92±2.16*	31.07±5.63*
IR+34 °C group	95.10±6.41	86.82±5.59*#	79.75±6.28*#	62.98±5.12*#	55.60±3.06*#
+dp/dtmax (mmHg/s)					
Sham group	2507.85±184.21	2551.55±124.63	2590.06±169.06	2552.78±252.18	2596.50±151.53
IR+37 °C group	2544.41±266.22	2123.36±53.75*	1670.41±141.44*	1211.70±103.49*	853.47±80.68*
IR+34 °C group	2627.31±117.92	2341.78±97.13*#	2044.39±229.02*#	1782.29±128.58*#	1612.65±181.64*#
**-**dp/dtmax (mmHg/s)					
Sham group	−2561.56±173.34	−2577.43±321.82	−2708.53±155.76	−2523.53±156.60	−2558.99±205.82
IR+37 °C group	−2615.31±198.22	−2001.98±90.76*	−1684.68±216.32*	−1195.16±84.50*	−858.79±44.99*
IR+34 °C group	−2549.33±244.64	−2265.11±300.49*#	−2026.90±244.30*#	−1809.55±73.73*#	−1531.06±152.22*#

^*^P < 0.05 VS Con group; ^#^P < 0.05 VS HR + 37 + Veh group

### MTH improves mitochondrial function and alleviates mitochondrial damage and oxidative stress response mediated by myocardial ischemia–reperfusion injury

**Fig.2:**
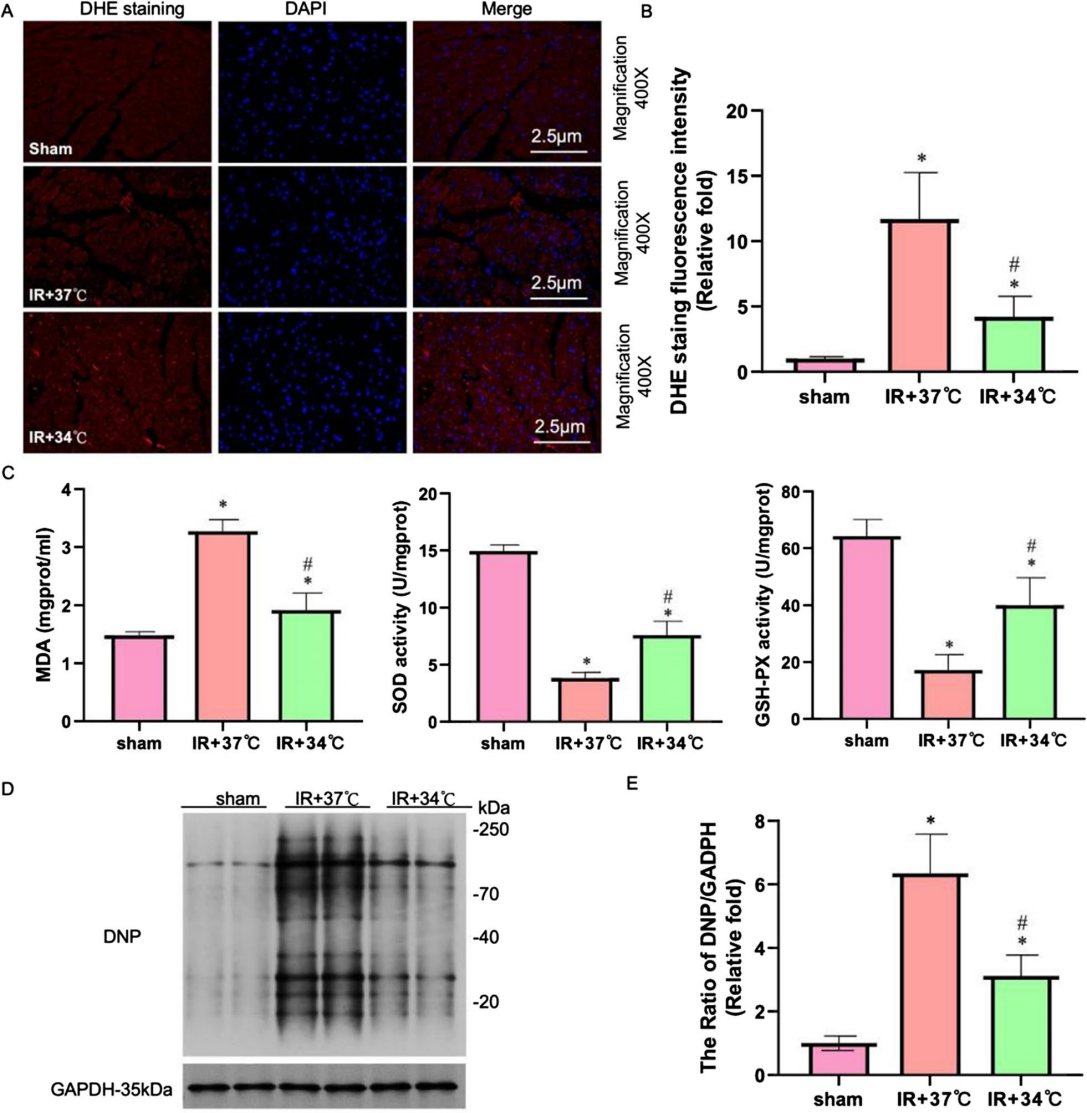
MTH inhibits oxidative stress response. **A**, **B** DHE staining and statistical graph. **C** Expression level of oxidative stress indicators (MDA, SOD, and GSH-PX). ^*^P < 0.05 VS sham group; ^#^P < 0.05 VS IR + 37 °C group

**Fig.3:**
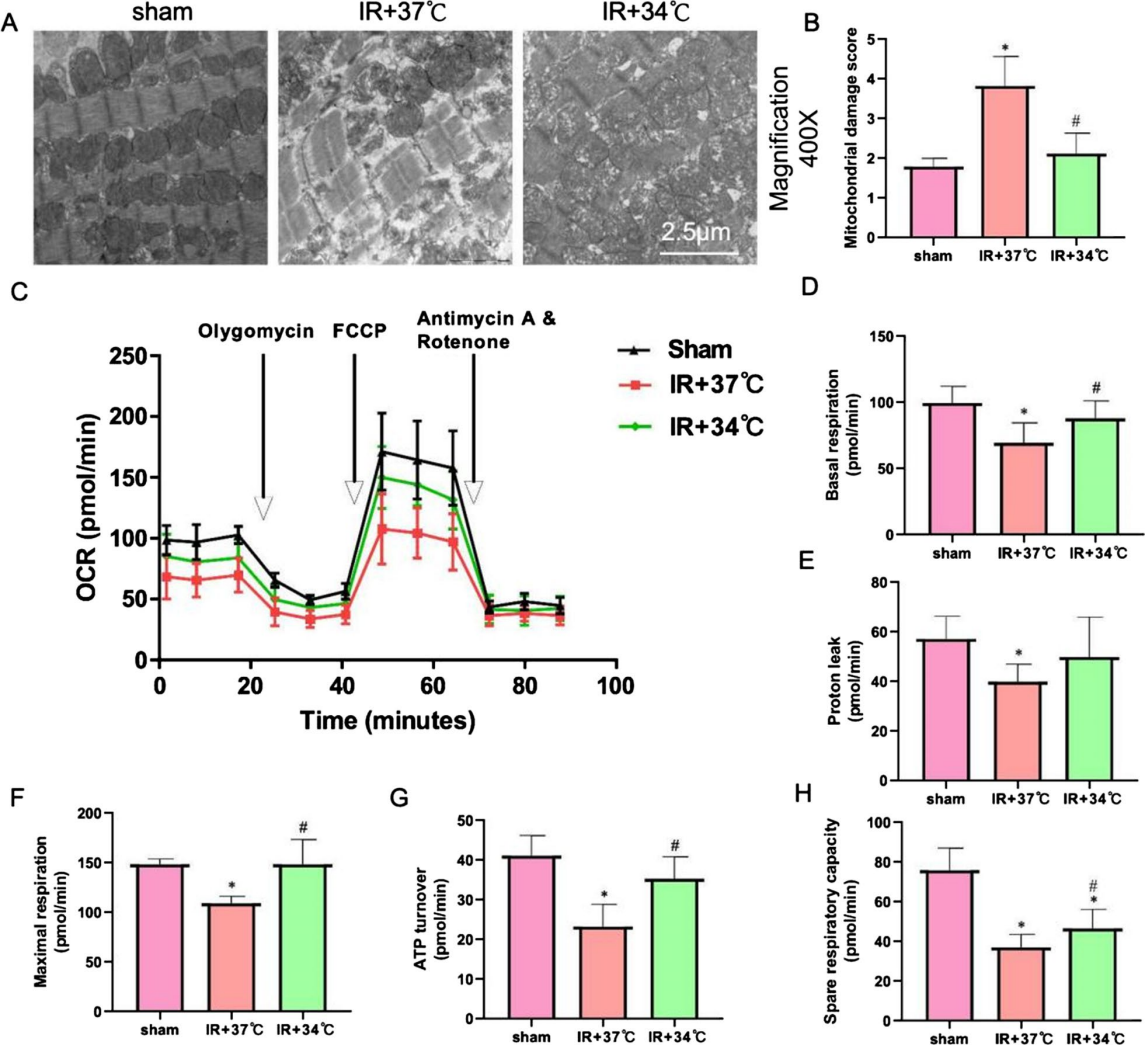
MTH reduces mitochondrial damage. **A** Mitochondrial structure observed under electron microscopy. **B** Mitochondrial damage score. **C**–**H** Mitochondrial function testing (OCR, Basal respiration, proton leak, Maximal respiration, ATP turnover, Spare respiratory capacity). ^*^P < 0.05 VS sham group; ^#^P < 0.05 VS IR + 37 °C group

Numerous studies have identified oxidative stress-induced mitochondrial damage as a key mechanism contributing to myocardial ischemia–reperfusion injury. In order to further investigate the relationship between the myocardial protective effect of MTH and oxidative stress, we measured oxidative stress indicators, mitochondrial damage, and mitochondrial function in rat myocardial tissues of each experimental group. The results indicated that the group exposed to IR + 37 °C had ten times higher ROS expression levels than the Sham group and 2–3 times higher levels than the IR + 34 °C group (Fig. 2A and B). Compared to the IR + 37 °C group, the expression levels of SOD and GSH in the IR + 34 °C group significantly increased, while the expression levels of MDA significantly decreased (Fig.2C, P < 0.05). Notably, the expression level of DNP in the IR + 37 °C group was six times higher than that of the Sham group and twice higher than that of the IR + 34 °C group (Fig.2D and E, P < 0.05). The above research results confirm that MTH can effectively improve oxidative stress damage mediated by myocardial ischemia–reperfusion injury. In order to further explore its mechanism, we observed the situation of mitochondria in the myocardial cells of rats in each group through the electron microscope. Electron microscopy observation revealed that compared to the IR + 37 °C group, the mitochondrial structure of the IR + 34 °C group was better protected, with a mitochondrial damage score similar to the Sham group (Fig. 3A, B). Meanwhile, Seahorse detection results showed that the mitochondrial function of the IR + 34 °C group was significantly improved, including basal respiration, maximum respiration, ATP production, and spare respiratory capacity, which were all superior to those of the IR + 37 °C group (Fig. 3C–H, P < 0.05). These results strongly suggest that MTH improves outcomes in myocardial ischemia–reperfusion injury by alleviating mitochondrial damage, enhancing mitochondrial function, and inhibiting oxidative stress damage.

### MTH induces an increase in OGT-dependent O-GlcNAcylation levels in myocardial tissue

**Fig.4:**
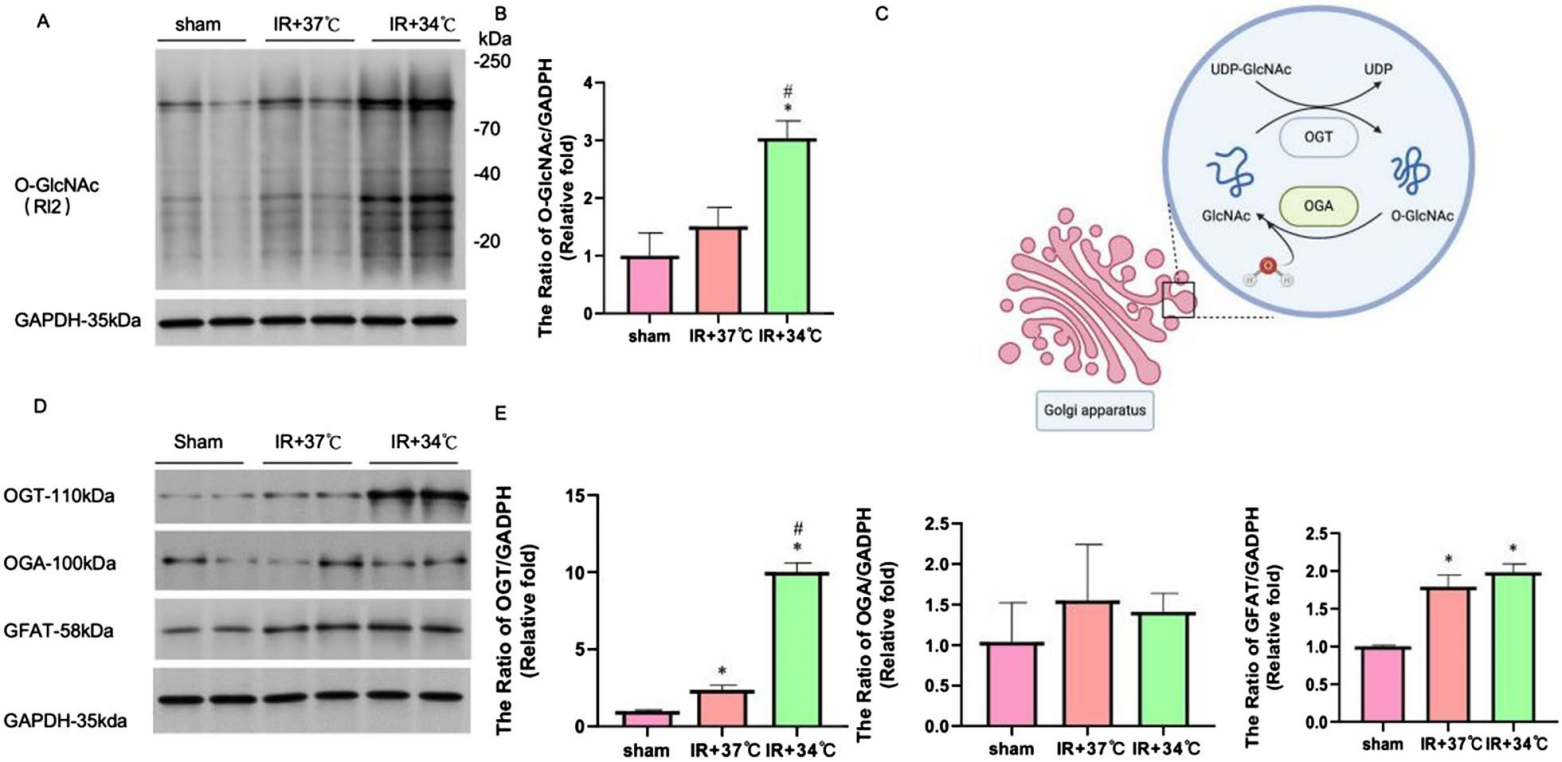
MTH affects the level of O-GlcNAcylation. **A** and **B** Expression level of O-GlcNAc. **C** O-GlcNAcylation process diagram. **D** and **E** Expression level of O-GlcNAcylation-related proteins (OGT, OGA, and GFAT). ^*^P < 0.05 VS sham group; ^#^P < 0.05 VS IR + 37 °C group

Research has confirmed that the O-GlcNAcylation level of myocardial mitochondrial proteins significantly affects mitochondrial function and oxidative stress levels (P < 0.05). Meanwhile, we have confirmed in previous studies that OGT-mediated O-GlcNAcylation plays a crucial role in myocardial ischemia–reperfusion injury [59] (Zhang et al., 2020). Based on this knowledge, we sought to investigate whether the myocardial protective effect of MTH is related to O-GlcNAcylation modification. According to the findings, the level of O-GlcNAcylation in the IR + 34 °C group was 3 × higher than Sham group and > 2 × higher than IR + 37 °C group. (see Fig. 4A and B). Furthermore, we further examine the expression levels of OGT, OGA, and GFAT in each group. We found that there was no significant difference in the expression level of OGA among the groups, and there was no significant difference in the expression level of GFAT between the two groups except for the Sham group. Simultaneously, it's important to highlight that the expression level of OGT in the IR + 34 °C group was markedly higher compared to the other two groups, as depicted in (Fig. 4D and E, P < 0.05). These experimental results strongly support our hypothesis that MTH exerts its myocardial protective effect through the modulation of O-GlcNAcylation.

### MTH upregulates the level of O-GlcNAcylation-modified COX10 and promotes its transfer to mitochondria

**Fig.5:**
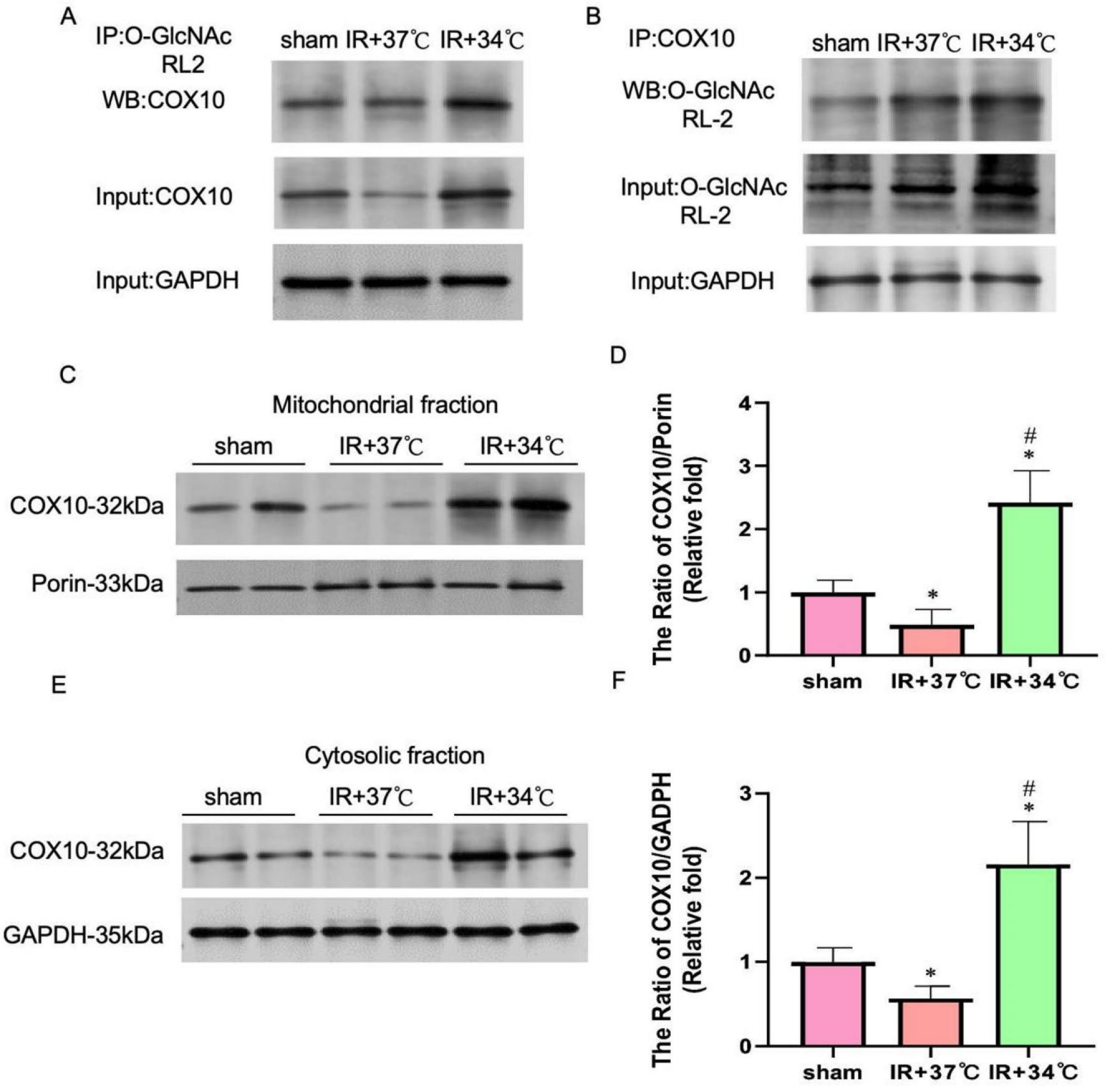
MTH upregulates the O-GlcNAcylation level of COX10 and promotes its expression. **A** and **B** The relationship between COX10 and O-GlcNAcylation. **C** and **D** Expression level of COX10 in mitochondria. E and F: Expression level of COX10 in cytoplasm. ^*^P < 0.05 VS sham group; ^#^P < 0.05 VS IR + 37 °C group

In order to further clarify the downstream targets of MTH regulating O-GlcNAcylation, we subsequently conducted immunoprecipitation experiments. Immunoprecipitation results showed that MTH could upregulate the O-GlcNAcylation level of COX10 (Fig. 5A–D). To further explore this mechanism, we specifically detected the expression levels of COX10 in the cytoplasm and nucleus. Experimental data showed that compared to the other two groups, the IR + 34 °C group exhibited significantly increased expression levels of COX10 in both cytoplasm and mitochondria (Fig. 5E–H, P < 0.05). Therefore, based on the experimental results presented in Sects. "MTH reduced myocardial infarction area and improved hemodynamic performance after ischemia–reperfusion injury in rats" to "MTH upregulates the level of O-GlcNAcylation-modified COX10 and promotes its transfer to mitochondria", we propose the following hypothesis: MTH improves mitochondrial function by regulating the O-GlcNAcylation level of COX10 and promoting its entry into mitochondria, thereby exerting its cardioprotective effects in myocardial ischemia–reperfusion injury.

### MTH promotes O-GlcNAcylation to improve hypoxia reoxygenation injury of cardiomyocytes

**Fig.6:**
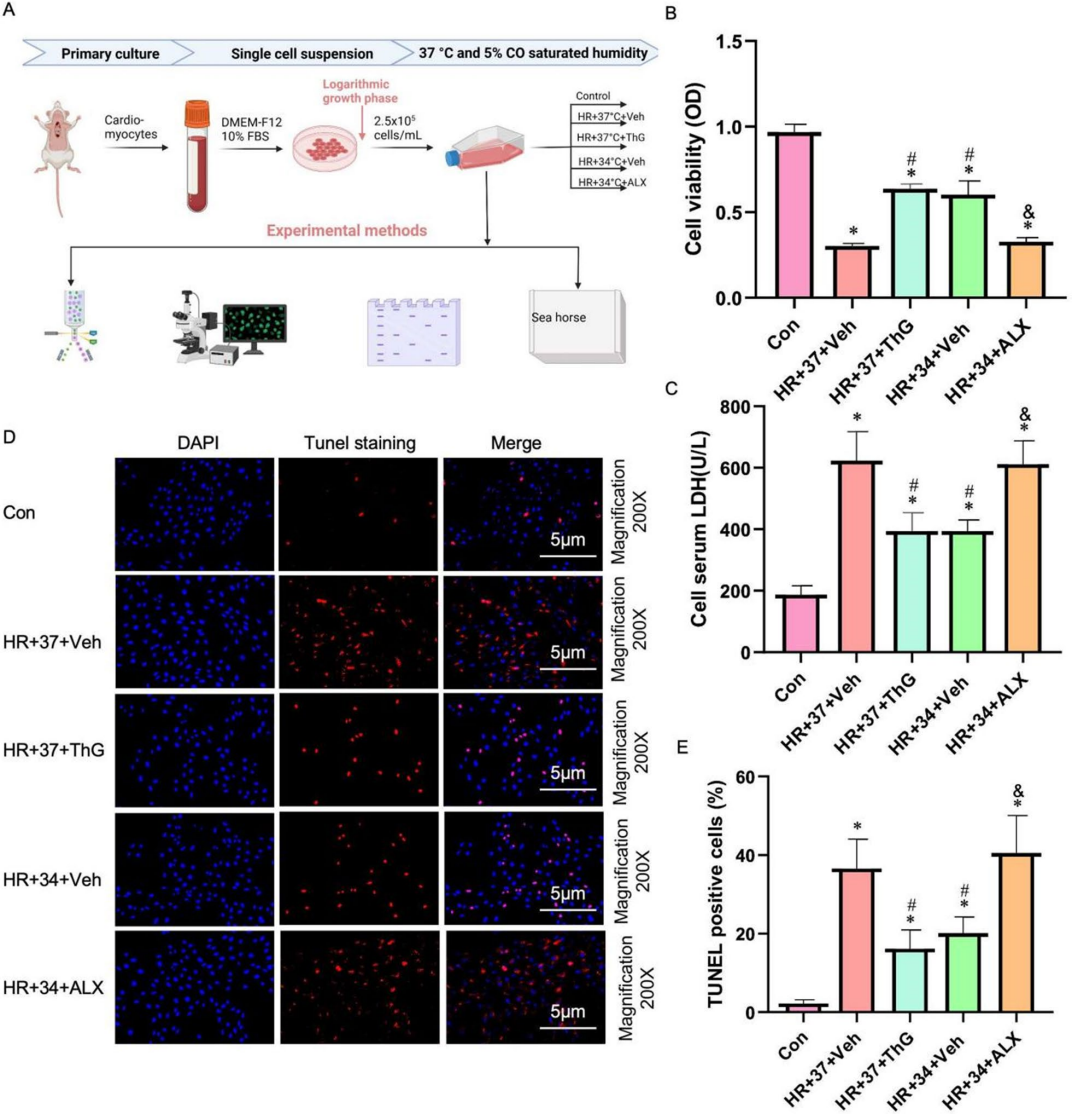
Cardiomyocyte viability, cell damage, and apoptosis rate. **A** Flow Chart of Cell Model Construction. B Cardiomyocyte viability. **C** Cell serum LDH. D AND **E** Tunnel staining and cell apoptosis rate. ^*^P < 0.05 VS Con group; ^#^P < 0.05 VS HR + 37 + Veh group; ^&^P < 0.05 VS HR + 34 + Veh group

To verify the hypothesis in Sect. "MTH upregulates the level of O-GlcNAcylation-modified COX10 and promotes its transfer to mitochondria", we constructed a cell hypoxia-reoxygenation (HR) model and intervened with corresponding drugs. Our observations revealed that cell viability in the HR + 37 + ThG and HR + 34 + Veh groups exhibited a significant increase compared to the HR + 37 + Veh group. Moreover, the instances of cell apoptosis and the expression levels of LDH were markedly reduced in the HR + 37 + ThG and HR + 34 + Veh groups when contrasted with the HR + 37 + Veh group (refer to Fig. 6B, C). These findings provide confirmation that ThG, an inhibitor of OGA, effectively enhanced O-GlcNAcylation and ameliorated myocardial cell injury. In addition, at low temperature (34 °C), the addition of ALX, an OGT inhibitor, abolished the protective effect of MTH. Myocardial cell viability was significantly (P < 0.05) reduced, whereas the expression level of LDH and the proportion of apoptosis were significantly (P < 0.05) increased (Fig.B-E). These findings offer compelling evidence that the protective mechanism of MTH involves the regulation of O-GlcNAcylation levels, which are consistent with the results presented in Sects. "MTH reduced myocardial infarction area and improved hemodynamic performance after ischemia–reperfusion injury in rats" and "MTH induces an increase in OGT-dependent O-GlcNAcylation levels in myocardial tissue".

### MTH upregulates the O-GlcNAcylation level of COX10 and inhibits mitochondria injury mediated by myocardial ischemia–reperfusion injury

**Fig.7:**
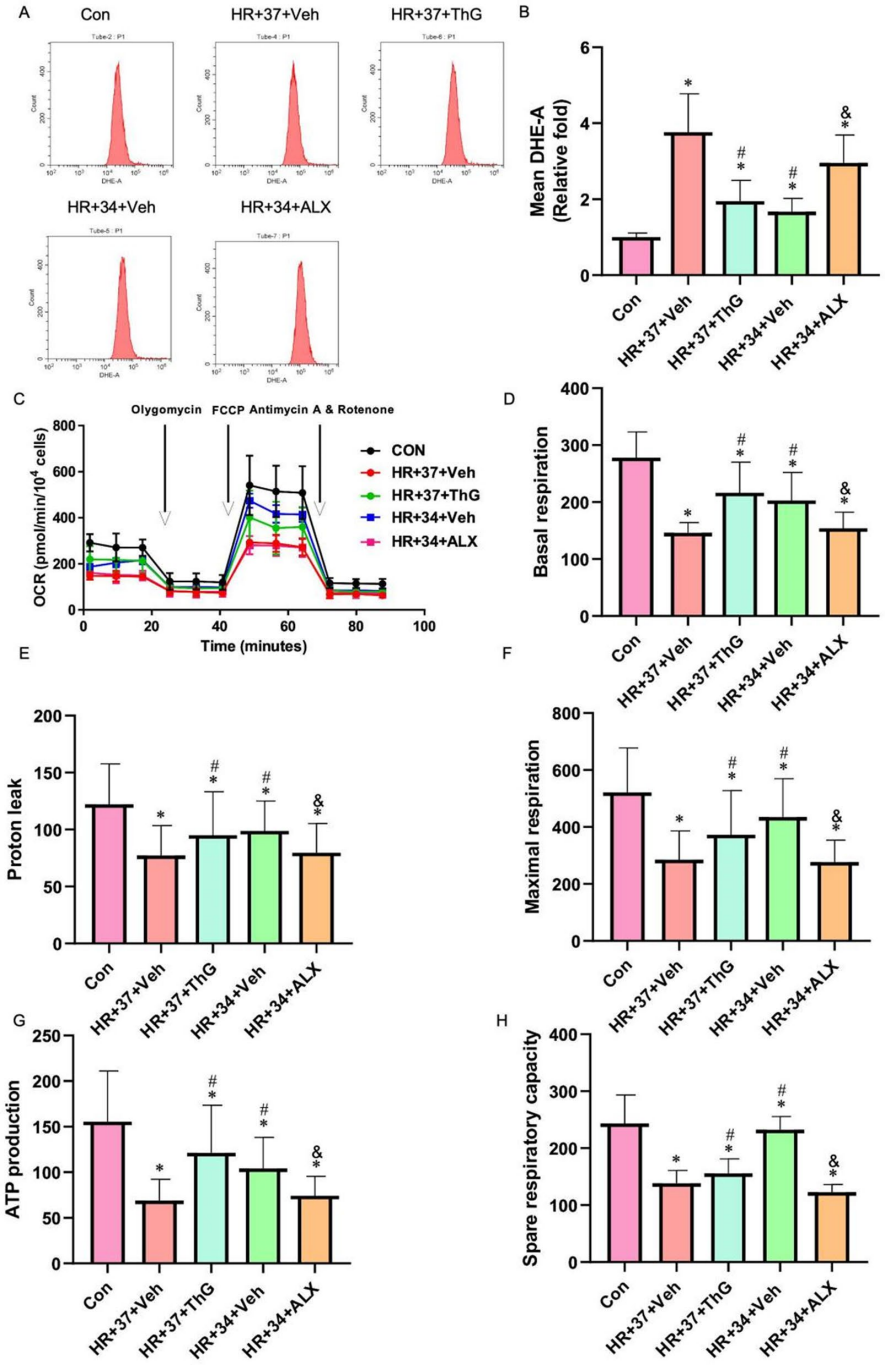
The regulation of oxidative stress and mitochondrial function by MTH is influenced by O-GlcNAcylation modifications. **A** and **B** Cell oxidation levels. **C**–**H** Mitochondrial function testing (OCR, Basal respiration, proton leak, Maximal respiration, ATP turnover, Spare respiratory capacity). ^*^P < 0.05 VS Con group; ^#^P < 0.05 VS HR + 37 + Veh group; ^&^P < 0.05 VS HR + 34 + Veh group

We further examined the expression levels of reactive oxygen species (ROS) and mitochondrial function in various cell groups to validate our hypothesis (Fig. 7). The introduction of the OGA inhibitor, ThG, at 37 °C, effectively suppressed ROS expression, and there were no noteworthy differences in ROS levels between the HR + 37 + ThG and HR + 34 + Veh groups. Meanwhile, at 34 °C, the addition of ALX promoted the expression of ROS and reduced the inhibitory effect of MTH on oxidative stress. Compared to HR + 34 + Veh, the ROS level in the HR + 34 + ALX group significantly increased (P < 0.05). Subsequently, we also tested the mitochondrial function of each group and found that ALX effectively abolished the protective effect of MTH on mitochondrial function. The basal respiration, maximum respiration, proton leak, ATP production, and spare respiration capacity in the HR + 34 + ALX group were significantly lower than those in the control group and HR + 34 + Veh group (P < 0.05).

**Fig.8:**
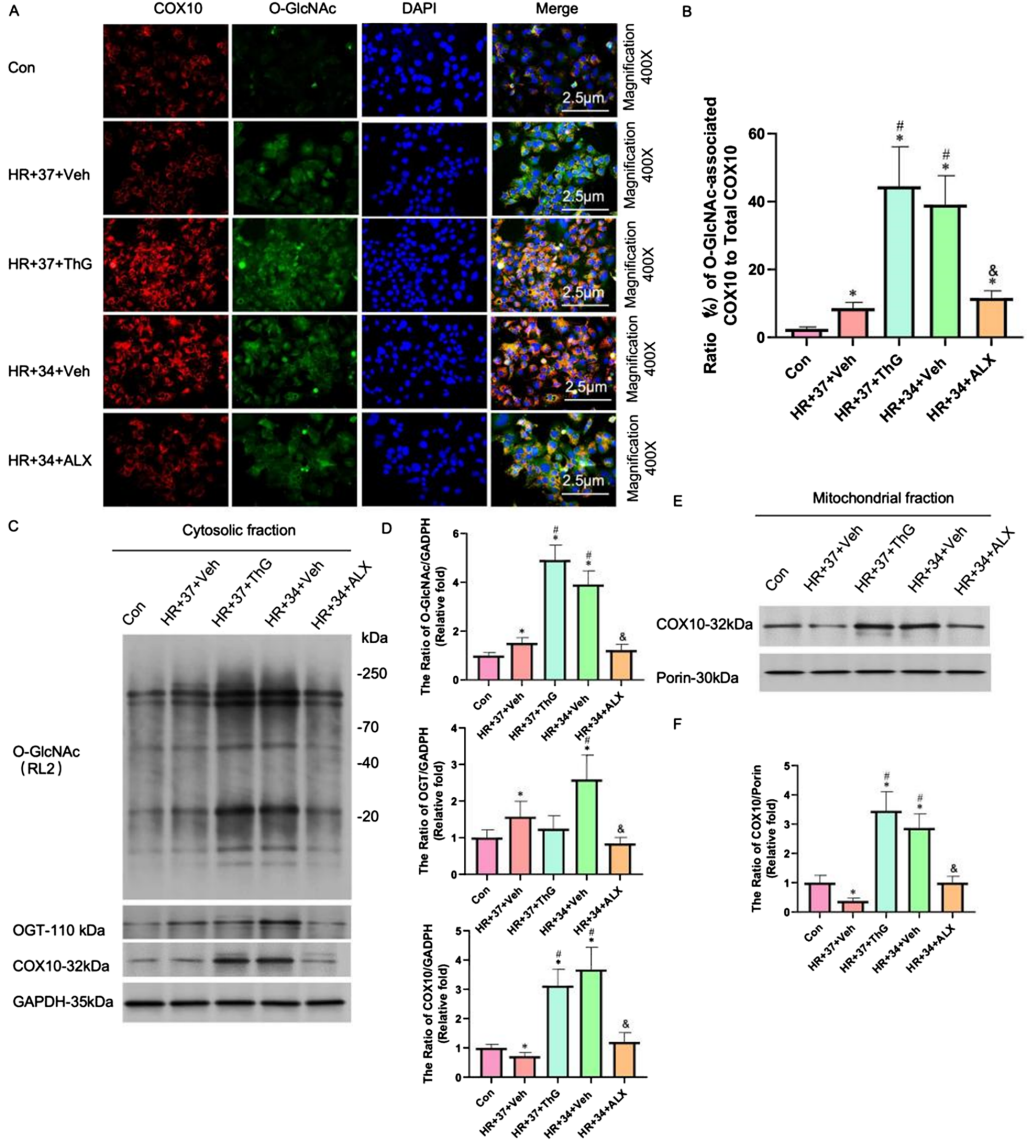
MTH promotes the expression of COX10 in cells and promotes the O-GlcNAcylation level of COX10. **A** and **B** Immunofluorescence and statistical images. **C** and **D** Expression levels of related proteins in the cytoplasm (O-GlcNAc, OGT-110, and COX10). **E** Expression level of cox10 in mitochondria. ^*^P < 0.05 VS Con group; ^#^P < 0.05 VS HR + 37 + Veh group; ^&^P < 0.05 VS HR + 34 + Veh group

In addition, immunofluorescence results supported our hypothesis, as the ratio of O-GlcNAc associated COX10 to total COX10 in the HR + 34 + Veh group was approximately four times higher than that in the HR + 37 + Veh group, and 2–3 times higher than that in the HR + 34 + ALX group (Fig. 8A, B). Notably, there was no significant difference in the ratio between the HR + 34 + Veh and HR + 37 + ThG groups (Fig. 8A, B). To further substantiate our hypothesis, we analyzed the expression levels of O-GlcNAcylation, OGT, and COX10 in the cytoplasm, in addition to evaluating COX10 expression within mitochondria (see Fig. 7C–F). The results showed that compared to the HR + 37 + Veh and HR + 34 + ALX groups, the expression levels of O-GlcNAcylation, OGT, and COX10 in the HR + 34 + Veh group were significantly increased (P < 0.05). It is worth noting that compared to the HR + 37 + Veh group, the expression levels of O-GlcNAcylation, OGT, and COX10 in the HR + 37 + ThG group also significantly increased (P < 0.05).

These thorough results demonstrated that MTH increases the expression of COX10 that has been modified by O-GlcNAcylation, reducing mitochondrial damage and dysfunction caused by myocardial ischemia–reperfusion injury. This mechanism ultimately contributes to the myocardial protective effects of MTH, which is consistent with the findings presented in Sects. "MTH reduced myocardial infarction area and improved hemodynamic performance after ischemia–reperfusion injury in rats" to "MTH upregulates the level of O-GlcNAcylation-modified COX10 and promotes its transfer to mitochondria".

## Discussion

Despite extensive research exploring different treatment options, myocardial ischemia–reperfusion injury is still a major concern in cardiovascular surgery. Numerous studies have revealed that several pathophysiological mechanisms contribute to this injury, including ferroptosis, calcium overload, inflammatory cascades, mitochondrial damage, and oxidative stress elicited by mitochondrial dysfunction [5, 29, 32],Mokhtari‐Zaer et al., 2019; [60]. Although clinical interventions like ischemic preconditioning (IPC) and pharmacological preconditioning (PPC) are employed, they have failed to deliver satisfactory outcomes in alleviating myocardial ischemia–reperfusion injury.

Therapeutic hypothermia is a method of controlling the body's temperature to achieve therapeutic goals. According to its different temperatures, it can be divided into deep hypothermia treatment (< 19.9 °C), moderate hypothermia treatment (20–27.9 °C), mild hypothermia treatment (27.9–31.9 °C), and MTH treatment (32–35 °C). Initially, mild hypothermia was used in neurosurgery to safeguard cranial nerves [14]. However, an increasing number of clinical studies have shown that mild hypothermia therapy also has a good protective effect on myocardial injury. It is worth noting that the 2021 International Guidelines for Cardiopulmonary Resuscitation state that all adult patients who recover autonomous circulation after cardiac arrest (i.e. lack meaningful response to language commands) should adopt Target Temperature Management (TTM), with a target temperature selected between 32 °C and 36 °C and maintained for at least 24 h. In the latest American Heart Association guidelines for cardiopulmonary resuscitation and emergency cardiovascular care, it is stated that all adults who do not follow instructions after return of spontaneous circulation (ROSC) should undergo temperature control, and it was recommended to keep the temperature between 32 and 37.5 °C (Cw et al., 2023). Simultaneously, numerous fundamental studies have indicated that mild hypothermia therapy at 34 °C has demonstrated notable efficacy in mitigating injuries to various organs in diverse animal models [3, 27, 28, 47] (C et al., 2010; J. L et al., 2023; Sl and Ra, 2011). In this experiment, we validated the protective effects of MTH in animal models and cardiac cell cultures. Compared to normal body temperature, MTH effectively reduced myocardial infarct size and attenuated ischemia–reperfusion-mediated cardiac injury. It also inhibited the release of CK-MB and LDH, and biomarkers of myocardial damage, while enhancing hemodynamic function. Moreover, MTH improved cell viability, suppressed LDH leakage, and decreased cardiomyocyte apoptosis, underscoring its potential for safeguarding cardiac cells during ischemia–reperfusion injury.

An oxidative stress response is one of the main pathological mechanisms leading to myocardial ischemia and reperfusion injury, which causes irreversible myocardial cell damage and cardiac dysfunction through multiple pathways [26] (D. L et al., 2023). In addition, the high levels of ROS generated by oxidative stress reactions harm mitochondrial structure and function, aggravating the already existing damage to myocardial cells [54] (Y et al., 2021). Notably, studies have shown that 32 °C hypothermia inhibits ROS release, improves mitochondrial dysfunction, and thus exerts protective effects [39] (R et al., 2013). In line with these results, our study also discovered that the level of ROS expression in the IR + 37 °C group was tenfold higher compared to that of the Sham group and 2–threefold higher in comparison to the ROS levels in the IR + 34 °C group. In contrast to the IR + 37 °C group, the IR + 34 °C group exhibited notable upregulation in the expression levels of the antioxidant enzymes of SOD and GSH, accompanied by a significant downregulation in the expression levels of MDA. These analogous results were also observed in cell cultures, aligning with previous reports. Research has spotlighted biomolecular carbonylation as a cardinal biomarker of oxidative stress, with escalations indicating heightened oxidative stress. Therefore, we evaluated DNP levels to elucidate biomolecular carbonylation patterns in the experimental mice groups. Our results evidenced significantly lower DNP content in IR + 34 °C compared to IR + 37 °C.

It is worth noting that previous studies have confirmed that MTH can effectively improve myocardial ischemia–reperfusion injury. Indeed, the mechanism by which MTH ameliorates myocardial ischemia–reperfusion injury remains enigmatic. O-GlcNAcylation modification stands out as a pivotal post-transcriptional chemical alteration within organisms, and it plays a critical role in various biological processes. Glucose generates Uridine diphosphate N-acetylglucosamine (UDP-GlcNAc) via the hexosamine biosynthesis pathway when GFAT is present. Subsequently, OGT catalyzes the transfer of single deoxyacetylaminopyranosides in β-O-linkages from UDP-GlcNAc to serine or threonine residues, forming O-GlcNAcylation products [41]. Conversely, OGA removes O-GlcNAcylation moieties. Previous studies had confirmed that 10 μM ALX and 0.5 μM ThG can effectively regulate the O-GlcNAcylation process of protein [4, 12] (C et al., 2004; F et al., 2009; Ga et al., 2011). Consequently, in this experiment, we also selected OGT and OGA at the same concentration.

O-GlcNAcylation modification plays a crucial role in myocardial ischemia–reperfusion injury. Research has shown that hypoxia adaptation (HA) can improve myocardial ischemia–reperfusion injury by upregulating the level of O-GlcNAcylation [50] (W et al., 2021). Meanwhile, glutamine can exert its myocardial protective effect by promoting the O-GlcNAcylation level of proteins [23] (J et al., 2007). More importantly, an elevated level of protein O-GlcNAcylation has been associated with enhanced mitochondrial function and the mitigation of myocardial ischemia–reperfusion injury(V et al., 2008). Additionally, cold exposure stimulates OGT and OGA expression, alluding to potential temperature-sensitive regulation of O-GlcNAcylation [13, 56] (Y et al., 2022). Herein, we demonstrated that O-GlcNAc levels in IR + 34 °C group were three-fold higher versus that of the Sham group and over two-fold higher relative to the O-GlcNAc levels in IR + 37 °C group. Moreover, OGT expression was markedly upregulated in IR + 34 °C compared to other groups (P < 0.001), while ALX abolished MTH-mediated impacts on O-GlcNAcylation and OGT.

**Fig.9:**
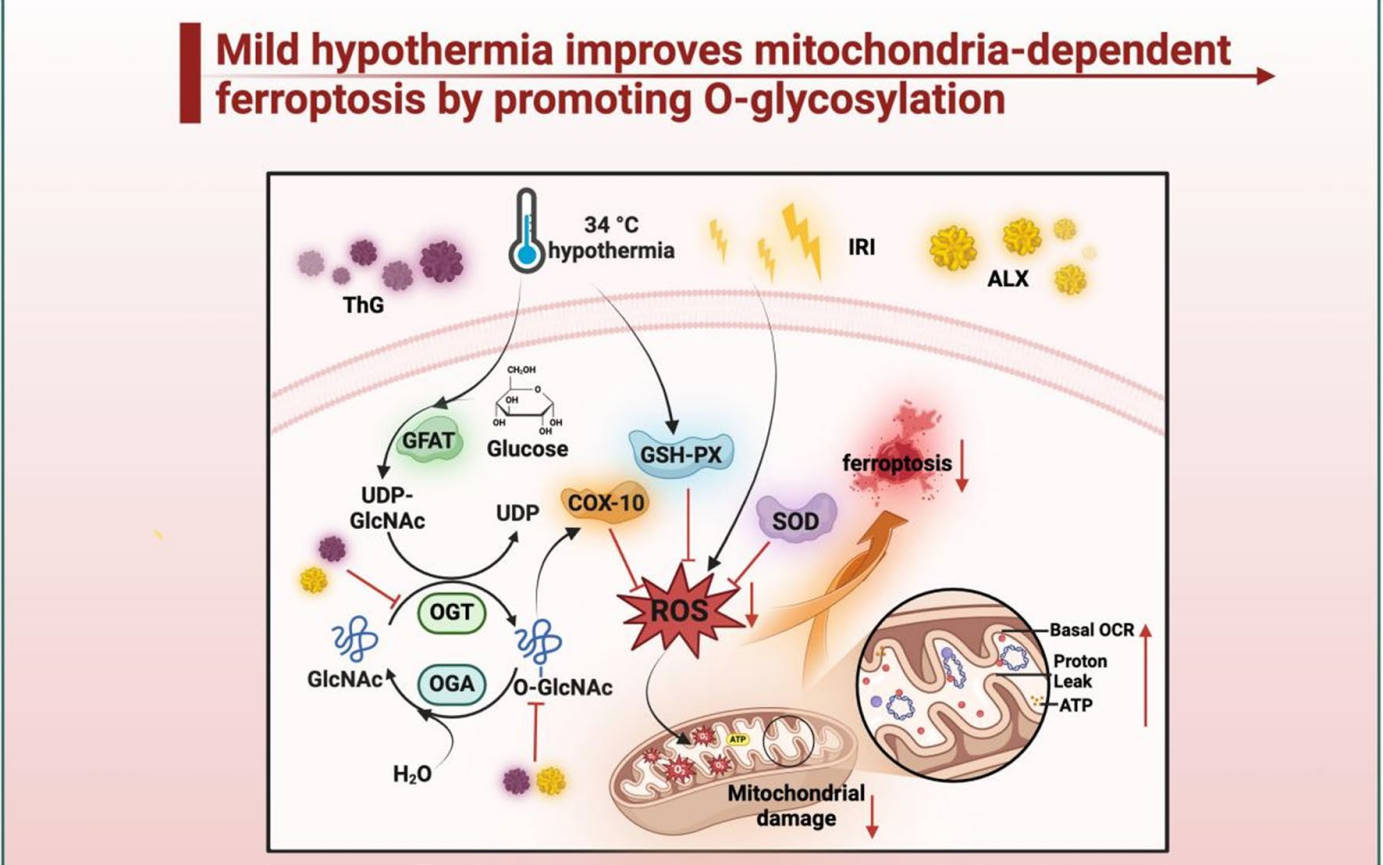
Mechanism diagram of MTH reducing myocardial ischemia–reperfusion injury

We also examined COX10, a pivotal enzyme in the mitochondrial electron transport chain, and its role in governing mitochondrial function. COX10 disruption directly impairs mitochondrial function, reducing ATP synthesis, depolarizing mitochondrial membrane potential, escalating reactive oxygen species production, triggering apoptotic signaling, and eliciting cell death. Recent research has underscored the importance of COX10 in mitochondrial cardiomyopathy [41] (A. S et al., 2022). In order to further clarify the relationship between O-GlcNAcylation and mitochondria, we found that MTH plays its role in myocardial protection by up-regulating the O-glycosylation level of COX10 and promoting its transfer to mitochondria. At the cellular level, our investigation revealed that ALX effectively prevented the alteration of COX10 through MTH-mediated O-GlcNAcylation, consequently obstructing the translocation of COX10 into mitochondria. In contrast, the inhibition of OGA by ThG significantly enhanced the localization of COX10 within the mitochondria. Cumulatively, these findings validate that MTH heightens COX10 O-GlcNAcylation and mitochondrial levels to enhance mitochondrial function, alleviate mitochondrial damage, suppress oxidative stress, and ultimately protect against myocardial ischemia–reperfusion injury, as shown in Fig. 9.

## Limitation

In this study, we only found that MTH affects the O-glycosylation level of COX10 at the animal and cellular levels. We did not use transgenic, plasmid transfection, or lentiviral interference techniques to directly manipulate the O-GlcNAcylation level of COX10, thereby confirming that MTH exerts myocardial protection by manipulating the O-GlcNAcylation level of COX10. Meanwhile, the detection of COX10 glycosylation sites is also a key focus of our subsequent experiments. We will analyze the O-GlcNAcylation sites of COX10 through mass spectrometry and further confirm our research results by constructing O-GlcNAcylation mutants of COX10. Importantly, this study provides a new perspective for enhancing hypothermic myocardial protection by improving mitochondrial function by regulating the O-GlcNAcylation level of COX10, thereby exerting the cardioprotective effect of hypothermia. Whether this mechanism can be further validated in clinical research in the future, and whether it can enhance the therapeutic effect of hypothermic myocardial protection by regulating COX10 glycosylation, is worth further research.

## Conclusion

In this study, we found that MTH can effectively alleviate oxidative stress damage, improve mitochondrial structure and function, and thus exert myocardial protective effects. Through further mechanism research, we have identified that MTH can alleviate mitochondrial damage induced by myocardial ischemia–reperfusion injury by upregulating the O-GlcNAcylation level of cytochrome C oxidase COX10 and promoting COX10 transfer into mitochondria. Importantly, O-GlcNAcylation plays a crucial role in mitochondria, and previous research has shown that O-glycosylation can affect mitochondrial autophagy by activating ferroptosis(F et al., 2022). Myocardial ischemia–reperfusion injury can be induced by ferroptosis, which is a key mechanism. Further exploration is needed to understand the protective mechanism of MTH against this injury. Our research findings provide a stronger theoretical basis for the perioperative use of MTH to prevent myocardial ischemia–reperfusion injury, and also provide new targets for the prevention and treatment of myocardial ischemia–reperfusion injury.

## Data Availability

The datasets used and/or analysed during the current study are available from the corresponding author on reasonable request.

## Abbreviations

ATP: Adenosine Triphosphate
COX: Cytochrome *c* oxidase
IP: Immunoprecipitation
LDH: Lactate dehydrogenase
IPC: Ischemic preconditioning
MTH: Mild therapeutic hypothermia
MIRI: Myocardial ischemia–reperfusion injury
OGA: O-GlcNAcase
OGT: O-GlcNAc transferase
PPC: Pharmacological preconditioning
PTM: Post-translational modification
ROS: Reactive oxygen species
